# Health professionals’ involvement in volunteering their professional skills: a scoping review

**DOI:** 10.3389/fmed.2024.1368661

**Published:** 2024-04-26

**Authors:** Ima Strkljevic, Anne Tiedemann, Juliana Souza de Oliveira, Abby Haynes, Cathie Sherrington

**Affiliations:** ^1^Institute for Musculoskeletal Health, The University of Sydney and Sydney Local Health District, Sydney, NSW, Australia; ^2^School of Public Health, Faculty of Medicine and Health, The University of Sydney, Sydney, NSW, Australia

**Keywords:** skilled volunteers, professional volunteers, volunteering, health professionals, health promotion

## Abstract

**Background:**

Volunteering positively affects overall health of both volunteers and recipients through social interaction, support and physical activity. Health professionals’ volunteering has considerable potential to improve health outcomes in communities.

**Objectives:**

This study aimed to summarize published scientific literature regarding volunteering by health professionals.

**Method:**

Medine, Embase, Scopus, PsycINFO and CINAHLdatabases were searched to identify eligible studies published between 2010 and 2023. Data on study methods and findings were extracted and synthesized.

**Results:**

Of the 144 eligible studies, 80 (56%) used quantitative methods, 46 (32%) used qualitative, 18 (12%) used mixed methods and 8 (6%) were interventional. Doctors (74 studies, 51%) and nurses (*n* = 40, 28%) were the professions with most reports of volunteering. Half the studies were from USA (*n* = 77, 53%), followed by UK (*n* = 19, 13%), Canada (*n* = 12, 8%), and Australia/New Zealand (*n* = 11, 8%). International volunteering in low-to-middle-income countries was reported in 64 studies (44%). Providing service and training were the dominant types of activities (*n* = 90, 62.5%), with health promotion reported in only 4 studies (3%). Studies reported positive impact from volunteering, both professionally and personally. Time and family commitments were the main barriers. Enablers, barriers and impact were summarized in a socio-ecological map.

**Conclusion:**

Health professionals volunteer in diverse activities and report multifaceted benefits. Studies of volunteering interventions could enable new, sustainable approaches to health promotion.

## Introduction

Volunteering theory exists in many forms across different disciplines since the term itself has evolved over the decades and has embraced an array of activities (1). Despite conceptual complexities, volunteering theory conceptualizes volunteering as a complex, interdisciplinary, multidimensional, unique, and paradoxical phenomenon, which is commonly defined as unpaid work (1–3). The theory suggests that people generally work for gain or benefit, yet some offer their skills voluntarily when it is not biologically necessary, when they are not paid, without coercion and without clear benefits to their families. In economics, sociology, psychology and political science, volunteering is associated with social cohesion, welfare, citizenship, prosocial personality (4) and altruism (5). Examination of health professionals as volunteers could add a novel idiosyncratic dimension to the concept and theory of volunteering as demonstrated by Xu and colleagues (6), who captured the essence of nursing volunteering as “a process of serving others and improving one’s cycle of improving oneself through serving others, in turn achieving personal and professional growth.”

Volunteers are an invaluable asset to their communities as they provide their knowledge, skills, resources and time as an enormous, growing informal workforce, and remain engaged in their cause or mission for an extended period of time due to their prosocial behavior (7). Australia’s estimated 6 million volunteers (roughly 30% of the adult population) have proven extremely valuable during times of national crises involving destructive bushfires, droughts, floods, hurricanes and the unprecedented pandemic of COVID-19 (8). Apart from crises, volunteers dedicate their time to numerous regular activities related to sport, health, community and social welfare, all of which support social inclusion, education, help for marginalized groups, social connectivity and delivering public services (9).

Evidence supports the multiple positive effects of volunteering for the objective and subjective health of recipients and volunteers themselves (1, 10). Studies show that volunteering is associated with decreased mortality and improved physical health, mental health, social support and interaction, healthy behaviors and coping ability in volunteers. In recipients, volunteering can improve self-esteem, disease management, mental health, cognitive function, self-efficacy and years of life (1, 10). Engaging volunteers and middle aged or older adults in social interaction also reduces loneliness in volunteers, which is acknowledged as a modern-age epidemic associated with cardiovascular disease, obesity, dementia, depression, anxiety, a multitude of physiological disorders and even premature death (11–13).

The global aging population and increasing numbers of people with chronic disease puts strain on individuals, families and healthcare systems (14). Promotion of healthy aging should thus be a global priority. Physical activity in later life may prevent, or at least delay, the onset of age-related functional impairment (15); however, low rates of physical activity among older adults remains a major public health concern (16).

Volunteering by relevant health professionals, for example, physiotherapists or exercise physiologists, has enormous potential to contribute to scalable methods for promoting healthy aging including through physical activity. Consequently, it is important to identify new, low-cost, sustainable strategies for promoting physical activity and supporting middle-aged and older adults to enjoy active, independent and happy lives for as long as possible (15, 17, 18). Volunteering by health professionals could play an important role in these efforts for the general public and for specific groups.

This scoping review is the first review aimed to identify available published literature regarding involvement of health professionals in professional volunteering and to summarize data related to the following research questions:

1. What is the extent and type of available literature reporting participation of health professionals in professional volunteering?
2. What is the extent and type of professional volunteering by health professionals?
3. What are the characteristics of health professionals who volunteer their professional skills?
4. What is the impact of health professionals’ volunteering on:

- health professionals,
- recipients of volunteer assistance,
- society?

This study is important for (a) revealing gaps in the current knowledge base that may require further research, (b) identifying areas of health promotion that could benefit from health professionals’ volunteering, and (c) providing guidance as to the best strategies for attracting and sustaining health professionals to volunteer their professional skills.

## Methods

### Search strategy

We conducted a scoping review due to the variability of evidence and heterogeneity of data, methodology and disciplines involved in the relevant literature. The review was carried out according to PRISMA-ScR Protocol Checklist (19). Our protocol was prospectively registered with OSF (Open Science Framework) Registries on 10 June 2021.^1^ The review used mixed methods synthesis and was conducted in alignment with the Joanna Briggs Institute methodology for scoping reviews (20). The search and screening were conducted from 18 June 2021 until 19 October 2021. The search was updated in March 2023. Searches were performed in Medline, Embase, PsycINFO, Scopus and CINAHL for articles reporting studies of any design published in English language between January 2010 and April 2021. The search identified published research designed to study or evaluate volunteering of professional skills among health professionals. An example of the syntax is illustrated by the Medline search:

(volunteer* adj3 (nurs* or physician* or doctor* or practitioner* or therapist* or allied health or clinician* or optometrist* or dentist* or intern* or dietitian* or nutritionist* or physiotherapist* or exercise physiologist* or counsel* or health professional*)).mp.

We exported all initial records into Covidence whereupon we excluded duplicates and screened references, including title, abstract and full text.

### Study selection

As this scoping review focused on health professionals volunteering their professional skills in diverse settings and with diverse goals, our inclusion criteria were broad. We included various study designs, types of health professionals, and nature of volunteering activities. We considered as health professionals: medical staff (doctors, physicians, surgeons, specialists), dentists, a range of allied health professionals including physiotherapists and occupational therapists, nurses, psychologists/counselors, paramedics, pharmacists, dietitians, midwives, public health professionals and students of the included health professions.

Each title and abstract were independently screened against the inclusion criteria by two reviewers (IS and BSA), who then also assessed each retrieved full-text paper. Discrepancies that arose at either stage were resolved through discussions between these two reviewers and a third reviewer (JSO) who is a senior researcher with expertise in conducting reviews. Titles, abstract and full-text studies were screened using Covidence systematic review software (21).

### Selection criteria

Articles were included if they met the following criteria: English language in a peer-reviewed journal, and quantitative, qualitative or mixed method studies which offered sufficient data for analysis. Articles featuring conference abstracts, case studies, personal narratives, editorials, or protocols were excluded. Grey literature, unpublished data, abstracts, conference proceedings and articles published in professional bulletins were also excluded. Articles concerning health professionals volunteering as participants in a study or trial were also excluded as this scoping review focused specifically on volunteering of professional skills in ongoing community-based activities. Combinations of two or more health professions and/or students were included. Studies of health professionals’ volunteering but not using their professional skills were excluded. The following health workers were excluded: alternative medicine practitioners, naturopaths, community health workers in low-to-middle income countries (who are not qualified health professionals), peer volunteers, and counselors who are not qualified psychologists. The inclusion/exclusion criteria are outlined in Appendix 1.

### Data extraction and analysis

Data extraction was conducted by author IS using a standardized data extraction form developed by the team. Extracted information included: author, publication year, study characteristics (type of study, study design, country of study), country of volunteering, health profession involved, volunteering activity, duration and frequency of volunteering, mode of delivery, type of service provided, the setting, and individual level factors associated with volunteering (age, gender, years of professional experience, volunteering experience). The included qualitative and mixed methods articles were analyzed thematically to report the main findings on the impact of volunteering on health professionals, recipients and society relevant to research question four. The themes were coded as motivators, benefits, barriers and challenges of volunteering. Data coding and analysis was performed in Microsoft Excel by synthesizing the relevant data in a matrix with the thematic codes along the top and each study in a separate row. This form of framework coding allowed us to categorize the voluminous qualitative data and assign categories/codes systematically (22).

## Results

### The flow of studies through the review

**Figure 1:**
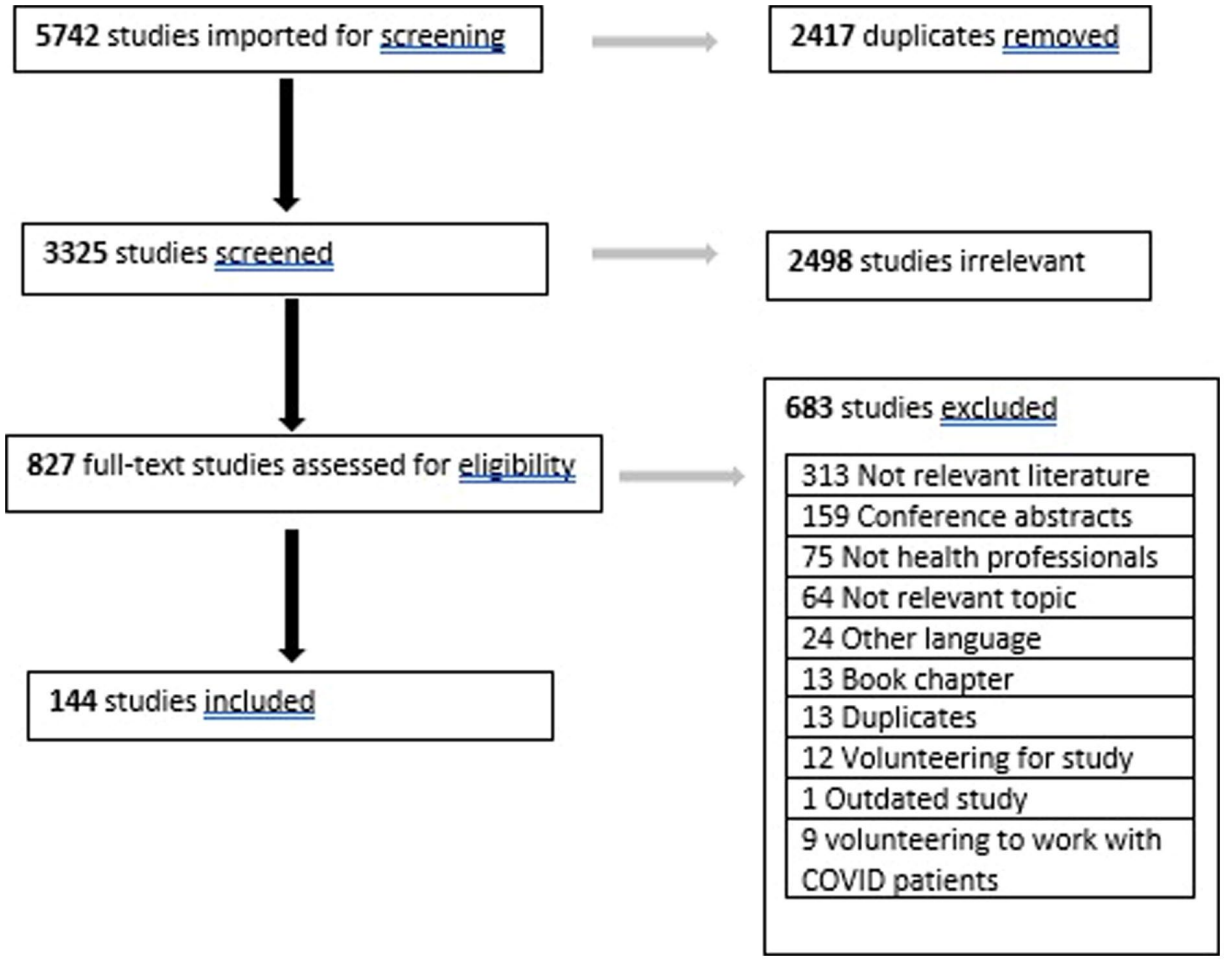
Literature search process.

The search yielded 5,742 records, with 3,325 studies remaining after removal of duplicates. After title and abstract screening 827 records remained, reducing to 144 after full-text screening (Figure 1).

#### Extent and type of available literature

Table 1 provides a summary of the descriptive statistics related to first research question, which focuses on the extent and type of literature available on health professionals volunteering their professional skills. The 144 studies that satisfied the inclusion criteria were summarized in terms of their total number, type of study, country of study and country of volunteering. This scoping review found that most of the scientific peer-reviewed literature on by health professionals volunteering their professional skills is quantitative (*n* = 80, 56%), qualitative (*n* = 46, 32%) or mixed methods (*n* = 18, 13%). Of the 80 quantitative studies, most were descriptive (90%), including surveys 68% (*n* = 54/80), literature reviews 6% (*n* = 5/80), systematic reviews 6% (*n* = 5/80), and other study types 10% (*n* = 8/80). Interventional research was reported in the remaining 10% of studies (*n* = 8/80), including two randomized controlled trials (3%) and six pilot trials (8%) (Table 1).

**Table 1:** Type of 144 included studies and type of health professionals (HP) and volunteering activities reported in the included studies.

Study design	*n* (%)
Quantitative study design	80 (56)
Survey	54 (38)
Systematic review	5 (3)
Randomized controlled trial	2 (1)
Pilot	6 (4)
Other descriptive	8 (6)
Literature review	5 (3)
Qualitative study design	46 (32)
Interviews	32 (22)
Open ended surveys	3 (2)
Reports analysis	2 (1)
Focus groups	4 (3)
Participatory action research	2 (1.4)
Qual systematic review	1 (0.7)
Ethnography	2 (1.4)
Mixed method studies	18 (13)
Surveys & interviews	13 (9)
Surveys & focus groups	4 (3)
Surveys & retrospective case log review	1 (1)
Volunteering health profession (HP) reported	*n* (%)
Doctors only	74 (51)
Nurses only	40 (28)
Other single HP	18 (13)
Dentists only	4 (3)
Psychologists only	8 (6)
Pharmacists only	1 (1)
Dieticians only	1 (1)
Midwives only	2 (1.4)
OTs only	1 (1)
PTs only	1 (1)
Multidisciplinary HP groups	19 (13)
Including doctors	13 (9)
Including nurses	11 (8)
Including pharmacists	4 (3)
Including allied HP (OT, PT)	4 (3)
Including dentists	3 (2)
Including psychologists	3 (2)
Including dietitians	1 (1)
Including public health HP	2 (1.4)
Unspecified HP	5 (3)
Students	49 (34)
Students & doctors	11 (8)
Students & nurses	6 (4)
Students & other HP	4 (3)
Students	49 (34)
Medical	20 (14)
Nursing	18 (13)
Health	5 (3)
Dietetics	1 (1)
Allied heath	3 (2)
Dentistry	4 (3)
Paramedic	2 (1.4)
Mental health	3 (2)
Type of volunteering activity	*n* (%)
Providing health service	39 (27)
Training/Education	28 (19)
Surgical	23 (16)
Disaster/Pandemic response	7/17 (5)/(12)
Community health	14 (10)
Counseling	7 (5)
Student-run clinics	7 (5)
Screening	7 (5)
Health promotion	4 (3)

Since some studies included volunteering activities from more than one category, the total amount of activities listed in table 6 (*n* = 153) is greater than the total number of studies (*n* = 144). HP, health professionals; OT, Occupational Therapist; PT, Physiotherapist.

Of the one-third (*n* = 46/144, 32%) of eligible studies that were qualitative, most used interviews (*n* = 32/46, 70%), followed by focus groups (*n* = 4/46, 9%) and open-ended surveys (*n* = 3/46, 7%). The remaining studies included reports analysis, ethnography and participatory action research (2/46 each, 4%) and one qualitative systematic review (*n* = 1/46, 2%).

The mixed method design was evidenced in 18 out of 144 (13%) identified studies where open-ended or closed-ended surveys were typically combined with interviews or focus groups. Of these 18 studies, there were 13 surveys combined with interviews (*n* = 13/18, 72%), 4 surveys with focus groups (*n* = 4/18, 22%) and 1 survey with retrospective case log review (*n* = 1/18, 5%). More details and summary graphs are shown in Appendix 2.

### Country of study

Most of the studies originated in the United States of America (USA, *n* = 77, 53%). The second and third largest contributors to the research about professional volunteering by health professionals were the United Kingdom (UK) (*n* = 19, 13%) and Canada (*n* = 12, 8%). Australia and New Zealand contributed 11 studies (7%). The remaining 25 studies (17%) were reported by researchers in 18 different countries (7 from Europe, 9 from Asia, 1 from South America and 1 from Africa). Geographic mapping of the countries which conducted studies on volunteering is provided in Appendix 3.

### Country of volunteering

Data regarding the country in which volunteering was undertaken described in the available literature were also analyzed and recorded (Appendix 4). There were 80 studies (56%) which reported domestic volunteering projects (i.e., the same country as the study report originated from) and 64 studies (44%) which described international volunteering activities and missions. Most of the studies reporting domestic volunteering were conducted in USA (*n* = 38, 26%), with 7 (5%) in UK, 7 (5%) in Canada, 5 (4%) in Australia and New Zealand, and the remaining 23 (16%) were from the 16 countries listed in Table 2 and Appendix 4. The reported international health professional volunteering projects (*n* = 64, 44%) were conducted in lower-to-middle income countries (LMIC) and 30 (21%) were multi-country projects. All LMICs of international volunteering are listed in Appendix 5. Studies from the USA reported most of the international volunteering projects (*n* = 38, 26%), followed by UK (*n* = 13, 9%), Canada (*n* = 5, 3%), Australia and New Zealand (*n* = 6, 4%), Germany and Brazil (*n* = 1, 0.7% each).

**Table 2:** Countries from which included studies originated and in which domestic, international and COVID-19 specific volunteering occurred.

Country	Origin of studies		Domestic volunteering		International volunteering		COVID volunteering	
	*n*	%	*n*	%	*n*	%	*n*	%
	144	100	80	56	64	44	20	14
USA	77	53	38	26	38	26	7	4
UK	19	13	7	5	13	9	1	0.7
Canada	12	8	7	5	5	3	0	0
Australia & NZ	11	7	5	3.5	6	4	0	0
Spain	3	2	3	2	0	0	1	0.7
China	3	2	3	1	0	0	1	0.7
Saudi Arabia	2	1	2	1	0	0	2	1
Germany	2	2	2	1	1	0.7	1	0.7
Thailand	2	1	2	1	0	0	1	0.7
South Africa	1	0.7	1	0.7	0	0	0	0
Brazil	1	0.7	1	0.7	0	0	0	0
Taiwan	1	0.7	0	0	1	0.7	0	0
Italy	1	0.7	1	1	0	0	1	0.7
Denmark	1	0.7	1	0.7	0	0	0	0
Indonesia	1	0.7	0	0	0	0	1	0.7
Ireland	1	0.7	1	0.7	0	0	0	0
Israel	1	0.7	1	0.7	0	0	0	0
Iran	1	0.7	1	0.7	0	0	1	0.7
Nepal	1	0.7	1	0.7	0	0	1	0.7
France	1	0.7	1	0.7	0	0	1	0.7
Georgia	1	0.7	1	0.7	0	0	0	0
Singapore	1	0.7	1	0.7	0	0	1	0.7

### Extent and type of volunteering

The health professional backgrounds of those reported in the literature as volunteering professional skills were medical, nurses, dentists, psychologists/counselors, pharmacists, dietitians, midwives, occupational therapists, physiotherapists, paramedics, public health professionals and students (Table 1; Appendix 6). Of all reported health professionals involved in professional volunteering, medical professionals, including doctors, physicians, surgeons and medical specialists, were the dominant profession (74 studies, 51%). Nurses, as traditionally involved professional volunteers, were the second highest contributor to volunteering of health professional skills reported in 40 studies (28%). Involvement of all other single health professions was reported in 18 studies (13%): dentists (*n* = 4, 3%), psychologists (*n* = 8, 6%), midwives (*n* = 2, 1.4%), pharmacists (*n* = 1, 1%), dietitians (*n* = 1, 1%), occupational therapists (*n* = 1, 0.7%) and physiotherapists (*n* = 1, 0.7%). Nineteen studies (13%) reported involvement of multiple health professions in a volunteering activity. The combined health professions reported were doctors (*n* = 13, 9%), nurses (*n* = 11, 8%), pharmacists (*n* = 4, 3%), allied health professionals (occupational therapists and physiotherapists) (*n* = 4, 3%), dentists (*n* = 3, 2%), psychologists (*n* = 3, 2%), public health professionals (*n* = 2, 1.4%), dietitians (*n* = 1, 1%) and 5 studies (4%) had not specified health professions involved in a volunteering project.

Trainee health professionals were reported as volunteers in 49 studies (34%) (Table 1; Appendix 7). They were medical students (*n* = 20, 14%), nursing (*n* = 18, 13%), health (*n* = 5, 3.5%), dentistry (*n* = 4, 3%), allied health (*n* = 3, 2%), mental health (*n* = 3, 2%), paramedic (*n* = 2, 1.4%), and dietetics (*n* = 1, 1%). Twenty-one studies (15%) recorded students participating in professional volunteering activities together with fully qualified health professionals [doctors (*n* = 11, 8%), nurses (*n* = 6, 4%)] and other health professionals (*n* = 4, 3%).

Most of the studies reported face-to-face volunteering activities (*n* = 116, 81%). Other modes of delivery included combination of face-to-face and online or phone delivery (*n* = 9, 6%), phone only (*n* = 6, 4%), online only (including teleconsultation and webchat) (*n* = 8, 6%), and phone or online (*n* = 3, 2%).

The frequency and length of volunteering activities were summarized in terms of continuous, sporadic or event-based engagement. For most international volunteering activities, the common format of engagement was a short-term medical mission with a duration of 2–8 weeks. Twenty studies (14%) reported international volunteering activities ranging from 1 week to 13 months in duration. Seventy-five percent of these reported missions ranged from 2–8 weeks. The duration of domestic health professional volunteering activities was reported in 25 studies (17%). This ranged from 1–2 weeks to 20 years (mean = 3.4 years, median 1.2 years). Domestic professional volunteering activities were predominantly continuous (*n* = 27/80, 34%), followed by event-based (*n* = 10, 13%) and sporadic (*n* = 4, 5%). Six studies (8%) did not report the regularity of volunteering engagement. Continuous engagement was taken as a prolonged, regular engagement with the activity on a weekly, monthly or other regularly scheduled basis. Sporadic engagement is taken as a long-term, occasional engagement. Event-based engagement is a one-off, planned event, such as health screening, training day, or a health promotion event in a community or health facility.

The categories of activities in which health professionals volunteered their professional skills are listed in Table 1. Provision of health services and care was the most frequent volunteering activity (*n* = 39), followed by training and education (*n* = 28) and conducting surgical procedures (*n* = 23). There were 24 volunteering initiatives related to disaster relief and emergency (*n* = 7) and pandemic response (*n* = 17). Community health initiatives (including placements and programs in various community groups such as youth, older men, alcohol and drug dependency, and homeless people) were identified in 14 studies, counseling in 7, student-run clinics in 7, health screening initiatives in 7 and health promotion programs in 4 studies on target groups such as culturally and linguistically diverse (CALD) groups, mothers with young children, and people with obesity, using the strategies of health education and lifestyle coaching.

### Characteristics of health professional volunteers

Addressing our third research question, we explored characteristics of volunteers reported in the identified studies as well as the facilitators, motivators and barriers of volunteering. Some studies reported proportions of surveyed health professionals currently involved in volunteering of professional skills (*n* = 45, 31%), the proportion of surveyed health professionals interested in future volunteering engagement (*n* = 22, 15%), gender (*n* = 23, 16%), race (*n* = 4, 3%) and age group (*n* = 15, 10%). Three studies (2%) reported the years of professional experience and professional role, which ranged from 1.15 years to 20 years (mean 3.35 [SD 5.1]). Within the studies that reported age (*n* = 15, 10%), the age of the volunteers ranged from 18 to 54 (Table 3).

**Table 3:** Characteristics of volunteers reported in included studies that surveyed health professionals.

Characteristics of volunteers reported in studies	Summary of characteristics reported in studies					Studies reporting the characteristic *n* (% of 144)
	Mean	SD	Median	IQR	Range	
Percentage of HPs reporting current volunteering	56.7	19.3	56.9	30.7	19.5–92.5	45 (31)
Percentage of HPs reporting interest in future volunteering	80.7	14.5	83	24.3	55.3–100	22 (15)
Percentage of females reporting volunteering	70.4	28.7	75	32	0–100	23 (16)
Percentage of Caucasians reporting volunteering	83.6	20.5	90.2	24.56	60.7–100	4 (3)
Reported years of volunteering	3.35	5.1	1.2	2.5	1.15–20	23 (16)
Predominant age group reporting volunteering	N/A	N/A	N/A	N/A	18–54 yrs	15 (10)

HP, health professional.

The proportion of survey respondents in individual studies who reported being currently involved in volunteering of professional skills ranged from 19.5 to 93%, with mean value of 57% and median 60%. The proportion of respondents interested in future volunteering ranged from 55 to 100% with a mean of 81% and median 83%.

In twenty of the twenty-three studies that reported gender of volunteers, the percentages of female participants ranged from 50 to 100%. Two studies, in which women were a minority group within the profession, reported 15 and 19% of female volunteers, and one study included only male nurses.

Fifteen studies (10%) reported the predominant volunteer age groups, ranging from 18 to 54 years (Appendix 8). Three studies (2%) reported the race of volunteers, which was mostly Caucasian, ranging from 61 to 100 percent (mean 84). Three studies (2%) reported statistics regarding the professional role of volunteers, indicating that 60% of volunteers were more likely to be in a clinical role compared to those in administrative or academic role, physicians (80%) compared to students (20%), and nurses and midwives (22 and 20% respectively) compared to allied health professionals and GPs (19% each). Six studies (4%) reported the volunteers’ years of professional experience and found that most were either at the beginning of their career with 1 to 5 years of professional experience (26%) or were experienced professionals with over 20 years of practice (32%).

### Motivators, facilitators, barriers, and impact

Extracted data were categorized as motivators, facilitators, barriers, benefits and limitations of volunteering, to address research question number 4: “What is the impact of health professionals’ volunteering on health professionals, recipients of volunteer assistance, and society?.” The qualitative and mixed methods studies identified in the review (*n* = 64, 45%) and a number of descriptive quantitative studies (*n* = 38, 26%) included reported themes regarding the mechanisms of engagement and the effects of health professional volunteering. These themes were also extracted and categorized with relevant sub-themes (Table 4).

**Table 4:** Themes related to motivators, facilitators, barriers and impact of health professional volunteering reported in 102 identified studies.^1^

Theme	Sub-theme	Study reference number
Motivators	Altruism/personal fulfilment/passion/drive	(23–46)
	Patriotism	(23, 47)
	Professional duty	(28, 35, 38, 41, 43, 44, 48–50)
	Reward	(42, 48, 51)
	Financial incentives	(41, 48),
	Certification opportunity/accreditation maintenance	(48, 52)
	Recognition	(48, 52, 53)
	Social interaction	(28, 54)
	Networking/professional development/employment	(30, 31, 36, 37, 44, 47, 49, 51, 55–57)
	Personal development	(30, 31)
	Travel/adventure/experience of new culture	(25, 32, 36, 38, 46, 58)
	Learning new skills	(24, 25, 36, 38, 41)
	Personal interest/history/culture/race/language/identity	(35, 40, 56, 59)
Facilitators	Younger age group	(60, 61)
	Clinical practitioners	(33, 60)
	2–4 weeks/year commitment	(60)
	Prior training	(41, 60, 62–64)
	Self-confidence; self-efficacy/leader	(27, 33, 47, 55, 56, 61)
	Good communicator/listener	(37)
	Flexibility	(27, 49, 65)
	Unselfishness/solidarity	(27, 65)
	Strong ethics/values	(27, 33, 39, 42, 65)
	Humility/compassion/empathy	(27, 33, 37, 47, 65)
	Previous volunteering experience	(25, 39, 53, 66–70)
	Academic support	(30, 62)
	Professional development CPD	(37, 47, 49)
	Resources	(30, 49, 62, 71)
Barriers	Personal health	(23)
	Availability/time	(23, 25, 26, 28, 30, 31, 39, 43, 48, 51, 60, 62, 68, 72–75)
	Transportation/distance	(23, 39, 60)
	Clinical commitments clashing with training sessions	(30, 72)
	Existing commitments (school, work, family responsibilities)	(23, 26, 28, 74, 76)
	Dissatisfaction with role of grading participants	(48)
	Lack of information	(26, 31, 62, 68, 71, 77)
	Lack of collaboration between institutions/inst. Support	(26, 43, 62)
	Perception of retired professionals/losing skills	(28)
	Uncertainty about roles	(24, 30, 31, 50)
	Lack of face-to-face contact	(40)
	Technology related issues	(78)
	Previous negative experience	(30, 31)
Benefits	Improved communication between providers and recipients	(28, 40, 64, 71, 76, 77, 79–82)
Impact on volunteer providers	Increased knowledge about CBP planning and implementation	(55, 76, 83, 84)
	Increased knowledge/skills, prof development	(28, 29, 31, 40, 45, 46, 49, 50, 55, 57, 67, 69, 72, 77, 79–81, 83, 85–95)
	Improved attitudes toward patient safety	(50, 72, 93, 95, 96)
	Positive personal experience	(29, 32, 35, 36, 39, 50, 52, 55, 67, 68, 70, 74, 77, 78, 80, 81, 83, 85, 86, 89, 96–101)
	Personal growth	(31, 41, 45, 55, 58, 67, 69, 71, 74, 79, 84, 87, 90, 92, 94, 102)
	Problem solving	(29, 83, 103)
	Reduced burnout	(92, 96, 104, 105)
	Increased compassion/empathy	(28, 55, 74, 83, 95, 96, 102)
	Rewarding/confidence	(32, 34, 40, 49, 79, 80, 82, 84, 88, 90, 92, 98, 100)
	Coping/adaptability	(92, 100, 106)
	Improved student mental health	(94)
Impact on recipient/society	Increased cultural competency	(40, 58, 64, 71, 79, 88, 90, 92)
	Increased programs nation-wide/world-wide	(56, 58, 89, 107–110)
	Support for individuals without insurance/health care/homeless	(56, 65, 75, 107, 111)
	Increased patient understanding/insight	(35, 55, 74, 79, 83, 89, 95, 96, 112)
	HP good role model	(28, 41, 44)
	Reduced morbidity & mortality	(94)
	Increased screening rate	(113, 114)
	Increased knowledge/skills	(32, 49, 57, 73, 82, 105, 108, 111, 115–118)
	Increased community confidence	(32, 37, 84, 116–119)
	Providing resources/high quality care	(33, 56, 89, 92, 99, 108, 111, 115, 119)
	Capacity building/sustainability/creativity	(56, 64, 82, 92)
	Gratitude/appreciation	(37, 54, 56, 86, 97, 120)
	Building relationships	(64, 71, 73, 82, 85, 89, 99, 115)
	Creating positive opportunities/spaces	(41, 94)
	Equitable health service/social justice/empowerment	(46, 75, 120)
	Inclusion, mindfulness, increased PA and improved body image	(118)
	Utilizing phone call to share information	(54)
Limitations	Crowding of local services	(27, 86, 87, 89, 90, 106)
	Inadequate follow-up	(25, 27, 32, 35, 39, 78, 87, 91)
	Reliance of foreign help/unsustainability	(27, 75, 89, 111, 114, 115)
	Dampening of investment in local health care	(27, 82, 87, 89, 115)
	Cultural/linguistic barriers	(32, 51, 57, 70, 87, 99, 108, 111, 121, 122)
	Poorly defined roles	(29, 85)
	Lack of training	(24, 29, 37, 50, 70, 85, 91, 112)
	Lack of venues for peer groups	(29)
	Poor volunteer acknowledgement/rewards	(29, 52)
	Unrealistic expectations from both sides	(32, 35, 37, 99, 111, 115, 122)
	Lack of resources/poor infrastructure	(39, 49, 57, 62, 75, 77, 90, 99, 103, 108, 121)
	Inadequate workload/rotations	(39, 49, 62)
	Anxiety/frustration/grief/loss of sense of purpose	(51, 62, 70, 86, 103, 106, 121, 123, 124)
	Adjusting to life after international placement	(121, 124)
	Poor coordination	(62, 108)
	Legal issues/liability	(109)
	Volunteer retention	(85)

^1^Color-coded themes used for the individual, interpersonal, community and societal levels of the socio-ecological map and for factors common for both community and societal levels; red-individual; yellow-interpersonal; blue-community; green-societal; grey-both community and societal levels.

**Figure 2:**
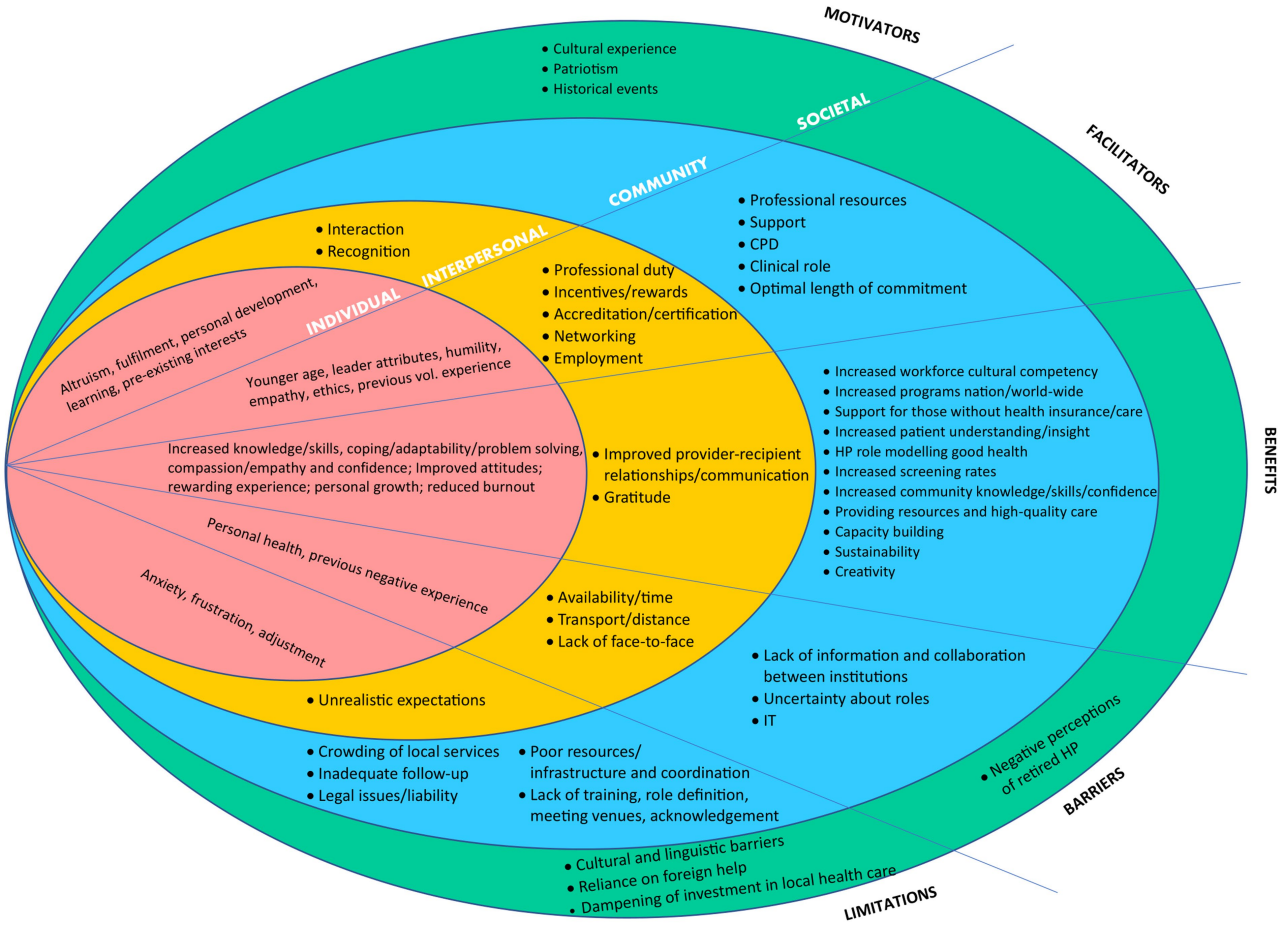
Socio-ecological map of health professional volunteering.

As the extracted themes in Table 4 suggested that volunteer health professionals act within a muti-layered social system, we applied a socioecological framework to the data. The socioecological framework was used as a guide to organize and summarize the evidence concerning various factors associated with health professionals’ volunteering and determinants of potentially promising recruitment interventions (125, 126). Despite the utility of socio-ecological framework to critically assess the factors related to volunteering by health professionals and to apply them as a foundation for adoption strategies in volunteering health interventions, we have not identified any studies which have systematically applied them in this topic. Figure 2 illustrates our findings that health professionals volunteer within a complex and dynamic environment that is a product of interactions, both positive and negative, occurring at four levels: individual, interpersonal (friends, family, peers, patients), community (health industry, professional organizations, institutions, volunteering organizations), and societal (social values, attitudes, customs, laws, culture) (125).

## Discussion

Given the increasing aging population coupled with the recent burden on public health and health services caused by the COVID-19 pandemic, we were particularly interested in investigating the potential of innovative forms of health promotion, in particular promotion of physical activity, to enable and support older adults to enjoy active, independent, and happy lives for as long as possible.

### The extent and type of professional volunteering in health professionals

This scoping review highlighted the dominant volunteering activities and health professions related to volunteering of professional skills. It also revealed the gaps in volunteering of certain health professionals as well as deficits in volunteered services provided by health professionals. The predominance of medical professionals and nurses in volunteering of professional skills by health professionals, is expected as they are the health professions which have traditionally provided primary care in emergencies, disaster responses, and numerous international and humanitarian missions (23, 127). The COVID-19 pandemic saw proliferation of mental and other health professionals’ involvement in volunteering through telehealth and online treatment (54, 85, 104, 128–131). The COVID-19 crisis has also shown that students of all health professions are a substantial and valuable force in stepping up and providing service where needed and are heavily involved in volunteering of professional skills as future health professionals (23, 24, 41, 42, 54, 86, 97, 132, 133). This potential should be harnessed in all spheres of health care and health promotion.

A key gap that this scoping review has revealed relates to the lack of reports of volunteering in health promotion by allied health professionals. Physiotherapists, for example, are uniquely placed as potential volunteers with skills to facilitate safe and effective programs to promote physical activity among middle-aged and older adults. Increased volunteering by physiotherapists and other allied health professionals could foster the development of new, sustainable, low-cost approaches for health promotion in the general public and for specific groups.

### The characteristics of health professional volunteers

There is evidence that many health professionals are involved in volunteering their professional skills, and studies report that more than half have previous volunteering experience (23, 53, 66, 127). Furthermore, a vast majority of health professionals studied have indicated an interest in future volunteering (23, 53, 127). This finding is encouraging when exploring the potential for volunteering among less studied health professions, such as physiotherapy or other allied health professions. As health professionals become involved in volunteering of professional skills, their engagement seems to last several years and may extend into retirement years. This is consistent with general volunteering, and not only limited to health professionals. Previous volunteering experience is a factor which makes health professionals likely to engage in volunteering in the future (25).

Gender is also a factor associated with volunteering in health professionals as women are heavily represented in studies as the main gender. It is known that women are more likely to participate in volunteering activities due to their higher levels of social interaction and engagement. Younger to middle age group is the age profile of a health professional volunteer (66), with health students in the early twenties being mostly engaged during COVID-19 pandemic (23). Studies suggest that being Caucasian, working in a clinical role, having under five or over twenty years’ experience, being married and having earnings in the highest bracket are characteristics of a typical volunteer (1, 7, 23).

### Socioecological view of health professionals’ volunteering

Our findings aligned with Omoto and Snyder’s (5, 134) Volunteer Process Model which highlights the antecedents (intrinsic factors, motivators and facilitators), experiences (satisfaction and involvement) and consequences (impact on community and society) of volunteering. Thus the impact of health professionals’ volunteering is multifaceted and can be influenced by motivators, barriers, benefits and limitations of volunteering across all layers of socioecological system from the individual and their immediate relationships to the health industry, and society as a whole (1, 2).

### Individual level

There are several perspectives on volunteering proposed from economic, psychological and sociological points of view, however the one that fits most closely with health discipline is the “public goods model” which rests on the assumption that volunteers are intrinsically motivated to donate their time to provide public services which they value, and to work for the benefit of others, including the desire to contribute to the wellbeing of the recipients (2, 135). This is evidenced by the most abundantly reported motivator for volunteering by health professionals, altruism or needing to help others, which is an ethical principle underpinning the clinical practice of all health professionals (2, 23–46). The commonality in health professions supports the potential for successful recruitment of health professionals as volunteers in variety of activities using strategies which appeal to altruism. Altruism, combined with empathy- a predictor of prosocial behavior, makes a great predictor of volunteering in a range of volunteering activities (5).

### Interpersonal level

Research has shown that volunteers whose volunteering expectations have been met and who are satisfied that their experience matches their motivations, are more likely to continue volunteering in the future (7, 25, 39, 53, 66–70). The presence of time conflicts between personal, professional and time spent volunteering has been identified as an important barrier to volunteering engagement and retention (7, 23, 25, 26, 28, 30, 31, 39, 43, 48, 51, 60, 62, 68, 72–75). Various organizational structures and processes, such as professional incentives, rewards, recognition, accreditation, networking and gratitude have been linked to volunteer behavior maintenance (7, 30, 31, 36, 37, 44, 47, 49, 51, 55–57). Thus, strategies to increase volunteering by health professionals might be most effective when employers and professional bodies play a role in recruitment and are actively supportive.

### Professional/community impact

When considering recruitment of health professionals as volunteers, the greatest barrier to volunteering, apart from time constraints and family commitments, is poor information about volunteering opportunities and lack of communication between volunteering and health organizations/affiliations (26, 31, 62, 68, 71, 77). Omoto and Snyder (7) suggest that simply having volunteers in one’s social network or being involved in one’s community is a reliable predictor of volunteer initiation. This suggests that strategies for increasing awareness within health professional organizations and providing a volunteer support infrastructure may be required. Peer-advocacy may be another useful strategy.

### Societal impact

The benefits of volunteering are evident not only on the individual level, in the volunteers and recipients, but in the wider community and society (7). This impact does not only reflect changes in attitudes and knowledge, improved health and subjective wellbeing, changes in health behavior, and professional and academic achievement (28, 29, 31, 40, 45, 46, 49, 50, 55, 57, 67, 69, 72, 77, 79–81, 83, 85–95, 136), but also in the establishment of community bonds, social networks, capacity building, sustainability, creativity, providing resources, support and growing nation-wide programs (56, 58, 75, 82, 92, 93, 107, 113, 114). Positive impacts of volunteerism at this macro level also include economic benefits and growth of social capital (7). Creating volunteer social networks and infrastructure at the organizational and societal level can increase the overall effectiveness of volunteers, as well as provides a means to recruit future volunteers (7).

### Suggestions for further studies

Qualitative research was highly represented in this scoping review as it provides insights into the mechanisms of volunteer engagement in particular settings, community groups or throughout the duration of the volunteering experience. However, quantitative research would benefit from more longitudinal designs to expand on the current, largely cross sectional, findings. In addition, more comparative intervention research is needed to provide insight into effectiveness, feasibility and sustainability of volunteering initiatives and programs.

### Strengths and limitations

This is the first scoping literature review to explore the extent of volunteering of professional skills among a range of health professionals in a variety of locations, settings, roles, types of activity and modes of delivery of volunteered service using different study designs. This review aimed to summarize the professional volunteering activities of health professionals, identify areas requiring attention, and provide a summary of the factors associated with volunteer engagement. The review encompassed both qualitative and quantitative studies to ensure a breadth of research was included. However, although the search strategy was comprehensive, it did not include grey literature or non-English language articles, which could pose a limitation. In addition, it is likely that there is a greater magnitude of health professionals’ involvement in volunteering of professional skills in wider range of areas which have not been reported in literature. The strength of this study lies in building a foundation for public health research reporting volunteering of health professionals as a means of health promotion.

## Conclusion

This study systematically identified and synthesized diverse research investigating volunteering of professional skills among health professionals. As the aging population continues to grow, it is important to explore different ways in which older adults, in particular, can be physically and socially active and maintain general wellbeing. This review highlights that health professionals have significant potential to be recruited as volunteers as they are highly motivated by altruism, professional development and personal growth. Volunteering activities need to be oriented more toward health promotion in older adults and other target groups rather than providing primary care as it has been the case thus far.

This scoping review revealed that allied health professionals are underreported as professional volunteers in the literature. Findings suggest they should be more utilized in volunteering activities as they have specialized health care knowledge and skills and are thus optimally placed to contribute expertise within health promotion initiatives, such as those targeting healthy aging. Physiotherapists and exercise physiologists in particular have an important role to play in the promotion of physical activity. It is recommended that both volunteering and professional organizations use this review to understand the determinants and trends of health professionals’ volunteering, by providing both social interaction and appropriate professional development opportunities or incentives. In addition, it is essential that organizations ensure that volunteering opportunities also involve older or retired health professionals, emeritus and student groups, who also have significant potential as health volunteers. Retired health volunteers may also reap the benefits of volunteering by continuing to be physically and socially active and engaged as they age (137).

## Author contributions

IS: Conceptualization, Writing – original draft, Writing – review & editing. AT: Supervision, Writing – review & editing. JS: Data curation, Supervision, Writing – review & editing. AH: Methodology, Supervision, Visualization, Writing – review & editing. CS: Conceptualization, Funding acquisition, Methodology, Supervision, Writing – review & editing.

## Footnotes

^1^ https://doi.org/10.17605/OSF.IO/M5QN4
