# Supplementary material for: Health professionals’ involvement in volunteering their professional skills: a scoping review
Source: Front Med (Lausanne). 2024 Apr 26;11:1368661. doi: 10.3389/fmed.2024.1368661 (PMC11082404; doi:10.3389/fmed.2024.1368661)
Supplement: Supplementary file 1 [file Data_Sheet_1.docx]

Supplementary Material

Table of Contents

Inclusion and exclusion criteria…………………………………………………………………………………………………..2

Type of study designs reporting health professional volunteering………………………………………..….…3

Geographic representation of health professional volunteering studies:

number of studies conducted by each country…………………………………………………………………………..4

The frequency of countries reporting domestic volunteering of professional skills in health professionals ………………………………………………………………………………………………………………………….....5

The list of reported Lower-to-Middle Income Countries (LMICs) of international volunteering by region………………………………………………………………………………………………………………………………………...6

Proportion of health professional volunteer types reported in the included studies…………… …...7

Proportion of student health professional volunteer types reported in the included studies…....8

Age range of volunteer health professionals across studies that reported age range……………..….9

# Appendix 1

**Inclusion and exclusion criteria**

| **Category** | **Inclusion** | **Exclusion** |
| --- | --- | --- |
| Publication language | Full text in English | Abstract-only, other languages |
| Publication date | Published after 01 January 2010 | Published before 01 January 2010 |
| Publication status | Published in peer-reviewed journals | Grey literature  Unpublished data  Abstracts  Conference proceedings  Professional bulletin articles |
| Study design | Literature review  Scoping reviews  Umbrella reviews  Systematic reviews  Experimental studies  Longitudinal studies  Qualitative studies/Interviews  Surveys/questionnaires  Qualitative studies/focus groups  Mixed methods | Conference abstracts  Narratives  Case studies  Protocols  Editorials |
| Populations | Volunteers:   - Allied health workers - Nurses - Dentists - Doctors - Humanitarian health workers - Students of included health professions - Retired health professionals | Health professionals in a volunteering role not using their professional skills.  Volunteers:   - Naturopaths - Alternative medicine practitioners - Community health workers - Peer volunteers - Counsellors who are not fully qualified psychologists - Health professionals volunteering in a study |

# Appendix 2

**Type of study designs reporting health professional volunteering**

# Appendix 3

**Geographic representation of health professional volunteering studies: number of studies conducted by each country**


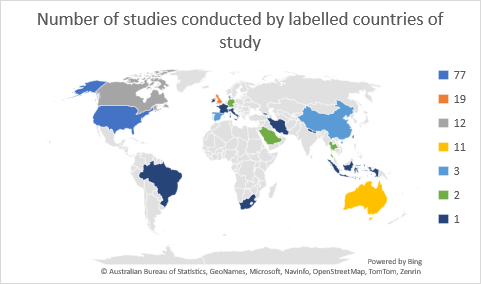


# Appendix 4

**The frequency of countries reporting domestic volunteering of professional skills in health professionals (n=80)**

# Appendix 5

**The list of reported Lower-to-Middle Income Countries (LMICs) of international volunteering by region**

|  | |
| --- | --- |
| **Central America** | Haiti |
|  | Dominican Republic |
|  | Trinidad and Tobago |
|  | Honduras |
|  | Nicaragua |
|  | Guatemala |
|  | Mexico |
|  | El Salvador |
| **South America** | Peru |
|  | Ecuador |
|  | Colombia |
|  | Bolivia |
| **Africa** | Botswana |
|  | Ghana |
|  | Uganda |
|  | Ethiopia |
|  | Tanzania |
|  | Sub-Saharan Africa |
|  | South Sudan |
|  | Sierra Leone |
|  | Malawi |
|  | Rwanda |
|  | Namibia |
|  | Kenya |
|  | Zambia |
|  | Nigeria |
|  | Zimbabwe |
| **Asia** | Nepal |
|  | Bangladesh |
|  | Cambodia |
|  | India |
|  | Pakistan |
|  | Vietnam |
|  | Bhutan |
|  | Myanmar |
|  | Malaysia |
|  | Afghanistan |
|  | Thailand |
|  | Mongolia |
|  | Philippines |
|  | Lebanon |

# Appendix 6

**Proportion of health professional volunteer types reported in the included studies**


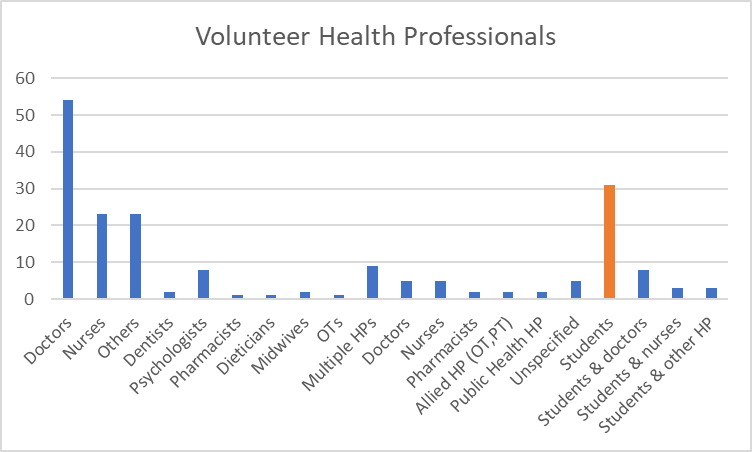


# Appendix 7

**Proportion of student health professional volunteer types reported in the included studies**


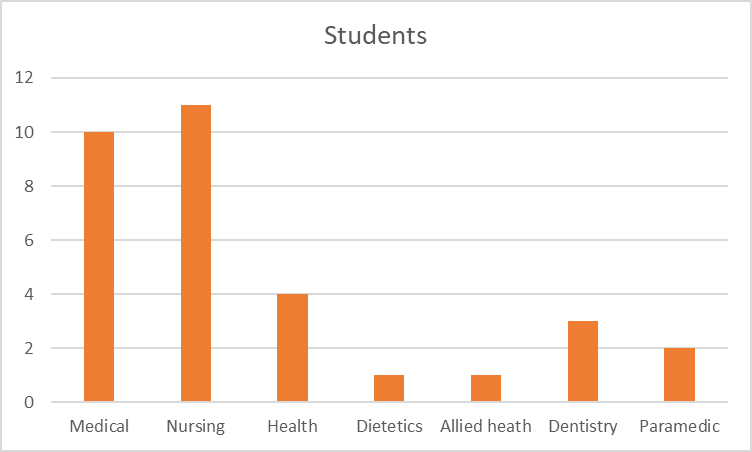


# Appendix 8

**Age range of volunteer health professionals across studies that reported age range**
